# Psychometric properties of a Thai version internet addiction test

**DOI:** 10.1186/s13104-018-3187-y

**Published:** 2018-01-24

**Authors:** Adam Neelapaijit, Manee Pinyopornpanish, Sutapat Simcharoen, Pimolpun Kuntawong, Nahathai Wongpakaran, Tinakon Wongpakaran

**Affiliations:** Department of Psychiatry, Faculty of Medicine, Chiang Mai University, 110 Intawaroros Rd., T. Sriphum, A. Muang, Chiang Mai, 50200 Thailand

**Keywords:** Internet, IAT, Loneliness, Psychometrics, Validity, Thai

## Abstract

**Objective:**

The aim was to assess the reliability and validity of a Thai version internet addiction test.

**Results:**

Cronbach’s alpha for the Thai version of the internet addiction test was 0.89. A three-factor model showed the best fit with the data for the whole sample, whereas the hypothesized six-factor model, as well as a unidimensional model of the internet addiction test, failed to demonstrate acceptable fit with the data. Three factors, namely functional impairment, withdrawal symptoms and loss of control, exhibited Cronbach’s alphas of 0.81, 0.81, and 0.70, respectively. Item 4, ‘to form new relationships with online users’, yielded the lowest loading coefficient of all items. Positive correlations between the internet addiction test and UCLA loneliness scores were found. The Thai version of the internet addiction test was considered reliable and valid, and has sufficient unidimensionality to calculate for total score in screening for excessive internet use.

**Electronic supplementary material:**

The online version of this article (10.1186/s13104-018-3187-y) contains supplementary material, which is available to authorized users.

## Introduction

Internet addiction has been specified as pathological internet use, compulsive Internet use and problematic Internet use [1, 2]. The inability to control Internet use impacts the body’s immune system, and work, social and academic performance [3–5]. Factors associated with Internet addiction include social isolation, low self-esteem, depression, personality traits, and interpersonal problems [6–8]. Despite the fact that problems arise from pathological use of the Internet, Internet addiction has not yet been included in the Diagnostic and Statistical Manual of Mental Disorders (DSM-5). The only behavioral addiction included in the latest version of the DSM is gambling disorder, which was included in a section recommending further study.

The prevalence of Internet addiction is estimated to be between 5.2 and 80.5% [9]. In Thailand, internet addiction has been investigated in both college and clinical settings, and 24.4% of participants were found to exhibit a low level of internet addiction [10, 11].

A number of instruments have been used to assess Internet addiction. The internet addiction test (IAT), developed by Young, is one of the most common tools and has been widely tested for its psychometric properties [12–16]. The IAT has demonstrated clinical relevance to addictive properties such as problems with time management, loss of control, impact on relationships [17]. The IAT has been shown to have excellent reliability, as assessed by internal consistency using Cronbach’s alpha, and good test–retest reliability [18]. Two studies from Korea, and one from Germany reported 2-week correlations ranging from 0.77 to 0.85 [18–20].

The validity of the test has been examined and reported in several languages including French [21], Italian [22], Portuguese [23], Spanish [16], German [24], Chinese [14], Malay [25], and Vietnamese [26]. The number of factors discovered through related research is inconsistent, ranging from 1 to 6 factors, depending on the sample and study setting employed [21, 22, 25, 27, 28]. Young proposed the six-factor model in the early stages of investigation of the IAT [28]. However, in a subsequent study with a larger sample size, the three-factor model was revealed to fit the data better [29]. Recent studies have found that two factors can adequately represent the model [12, 22]. The item commonly found to be misfitted is item 4, ‘to form new relationships with online users’, while other misfit items vary from study to study [18, 22].

While the IAT is increasingly being used to examine the Thai population, the Thai version of the IAT has never been examined for validity and reliability. The purpose of the present paper is to investigate the psychometric properties of the Thai version of the IAT in terms of factor structure, internal consistency, and possible misfit item(s) with other studies, as well as examining concurrent validity with other measurements.

## Main text

### Methods

#### Participants

First to sixth–year medical students from Chiang Mai University, Thailand, participated in this study by means of convenience sampling in 2015. Of 324 participants, 56.8% were female. The mean age was 20.88 (SD 1.8). All completed demographic data questionnaires and answered questions regarding their internet use using the IAT and the UCLA loneliness scale. The average time spent on the internet daily was 4.9 h (SD = 2.7), and 6.9 days per week. According to Young’s addiction level, 63.3%, none; 30.9%, mild; 5.2%, moderate; and 0.6%, severe.

#### Measurements

*The Thai version of the IAT*: The IAT, developed by Young [17], is a 20-item self-report instrument in which respondents rate their tendency for addiction to the internet using a five-point scale, ranging from 1 (rarely) to 5 (always). The IAT total score is the sum of the ratings given by the examinee in response to the 20 items. The higher the score, the higher the level of severity of internet compulsivity and addiction. Total scores of more than 30 are considered to indicate addiction. We obtained permission from Dr. Kimberly Young to translate the IAT and use it in our research study. The forward and backward translation process was done. The study sample had a Cronbach’s alpha of 0.89.

*UCLA loneliness scale*: The UCLA loneliness scale is an assessment tool used to screen and assess the severity of loneliness. It consists of 20 items asking respondents how often they experienced feelings of loneliness during the past week. Four-point responses to each item range from 0 (‘not at all’) to 3 (‘almost daily’). The Thai version demonstrated good reliability and validity [30]. The UCLA loneliness scale was used to evaluate the convergent validity of the Thai version of the IAT because a correlation between loneliness and internet addiction has been previously reported [31, 32].

#### Statistical analysis

Descriptive statistics was used for demographic data as well as data screening analysis for factor analysis. Item responses exhibited skewness and kurtosis (> ± 2) [33]. Exploratory factor analysis (EFA) using ordered categorical (ordinal) response was performed. For parameter estimation, as data were ordinals, robust weighted least square means and variance adjusted (WLSMV) were employed for estimators [34]. Confirmatory factor analysis (CFA) was used to test Young’s hypothesized six-factor model [28].

Regarding fit indexes, in the Comparative Fit Index (CFI) and Tucker–Lewis Index (TLI), a value > 0.90 indicates reasonable fit, and a value > 0.95 indicates good model fit; a weighted root-mean-square residual (WRMR) lower than 0.9, a root-mean-square error of approximation (RMSEA) of ≤ 0.06, and a standardized root mean square residual (SRMR) of < 0.08 indicate a reasonable fit [35–37]. In addition, the χ^2^ statistic has been used to test the goodness of model fit if the ratio χ^2^/*df* < 3 [33]. Missing data (0.5%) were replaced by series of means. Modification indices were added to the model after the initial analysis, and CFA was carried out using Mplus 7.4 [38]. Pearson’s correlation analyses were used to determine concurrent validity, and Cronbach’s alpha was calculated to assess internal consistency.

### Results

The objective of the analyses was to examine the internal consistency and the factor structure of the translated Thai version of the IAT.

Table 1 shows the mean and SD of each item. Cronbach’s alpha for all items was 0.89. The Corrected Item-Total Correlations were > 0.4, except for items 4 and 7, for which the alpha was estimated to be higher when either of these two items was deleted.

**Table 1:** Mean, SD, Cronbach’s alpha, and item-total correlation among IAT items

	Mean	SD	Corrected item-total correlation	Squared multiple correlation	Cronbach’s alpha if item deleted
1. Stay online longer than intended	3.25	0.996	0.438	0.343	0.884
2. Neglect household chores	2.41	1.149	0.496	0.407	0.883
3. Prefer excitement of internet	1.81	1.051	0.509	0.328	0.882
4. New relationships with online users	1.59	1.188	0.313	0.179	0.888
5. Others complain to you	1.2	1.052	0.553	0.361	0.881
6. Your work suffers	1.25	1.134	0.542	0.425	0.881
7. Check email before something else	1.03	1.23	0.363	0.235	0.887
8. Your job performance suffers	1.4	1.054	0.578	0.461	0.88
9. Become defensive when asked	0.74	0.918	0.496	0.351	0.882
10. Block out disturbing thoughts	1.09	1.128	0.536	0.345	0.881
11. Find yourself anticipating	1.56	1.104	0.563	0.417	0.881
12. Fear life without the internet	1.72	1.344	0.439	0.357	0.885
13. Snap if someone bothers	0.46	0.808	0.52	0.372	0.882
14. Lose sleep due to being online	2.24	1.289	0.522	0.353	0.882
15. Feel preoccupied with the internet	0.67	0.964	0.56	0.444	0.881
16. “Just a few more minutes”	1.73	1.444	0.482	0.382	0.884
17. Try to cut down the amount of time	1.78	1.231	0.583	0.479	0.88
18. Try to hide how long	0.67	0.946	0.56	0.377	0.881
19. Prefer spending time online	1.49	1.235	0.505	0.317	0.882
20. Feel depressed, when off-line	0.75	1.03	0.528	0.42	0.881

The eigenvalues for the sample correlation matrix (EFA) were 7.811, 1.723, and 1.269. The factor loading coefficients are set out in detail in Table 2. The scree plot suggested three factors due to the manner in which the slope levels off. The scree plot according to eigenvalues (Additional file 1: Figure S1) determined the number of these three factors (components).

**Table 2:** Cronbach’s alpha and loading coefficients of one-factor and 3-factor solutions of the Thai version of the IAT

IAT item	One-factor	Component		
		F1_functional impairment	F2_withdrawal symptom	F3_loss control
8. Your job performance suffers	0.678	0.743	0.449	0.283
6. Your work suffers	0.643	0.717	0.391	0.330
5. Others complain to you	0.628	0.658	0.497	0.099
2. Neglect household chores	0.629	0.650	0.359	0.419
9. Become defensive when asked	0.598	0.630	0.531	− 0.087
10. Block out disturbing thoughts	0.628	0.619	0.546	0.056
1. Stay online longer than intended	0.538	0.549	0.282	0.468
3. Prefer excitement of internet	0.605	0.546	0.496	0.240
7. Check email before something else	0.439	0.472	0.335	0.079
15. Feel preoccupied with the internet	0.694	0.446	0.778	0.080
20. Feel depressed, when off-line	0.652	0.461	0.750	− 0.070
13. Snap if someone bothers you	0.655	0.474	0.719	0.003
11. Find yourself anticipating	0.652	0.436	0.711	0.114
18. Try to hide length of internet use	0.668	0.538	0.666	0.075
12. Fear life without the internet	0.520	0.297	0.647	− 0.043
19. Prefer spending time online	0.583	0.499	0.562	0.052
14. Lose sleep due to being online	0.608	0.436	0.538	0.365
4. New relationships with online users	0.359	0.364	0.390	− 0.198
17. Try to cut down the amount of time	0.700	0.513	0.535	0.599
16. “Just a few more minutes”	0.589	0.364	0.472	0.592
Cronbach’s alpha	0.89	0.81	0.81	0.70

Cross-loadings (≥ 0.4) were found in all items except for items 6, 8 and 12. Item 4 had the lowest loading coefficient (0.356), followed by item 7 (0.439) on the one-factor model. Factor 1 covered items describing performance problems at school or at work, and relationship problems due to excessive Internet use. Factor 2, which dealt with ‘withdrawal symptoms’, involved depression, irritability, and fear when not using the Internet. Factor 3, ‘loss of control’, contained items about failed attempts to cut down on Internet use. The correlation coefficients between Factors 1 and 2, 1 and 3 and 2 and 3 were 0.594 (p < .05); 0.175 (p > .05) and 0.014 (p > .05), respectively. The Cronbach’s alphas for the factors were 0.70–0.81, and 0.89 for all items. The total scores of the Thai version of the IAT were significantly correlated with the UCLA loneliness scale (r = 0.293, p < .001). Level of loneliness, which is related to Internet addiction, was also significantly correlated, which provides strong support for the concurrent validity of the Thai version of the IAT.

Table 3 shows the goodness of fit of various models. The unidimensional models demonstrated poor fit, whereas the hypothesized six-factor models yielded better fit but were still unacceptable. The three-factor model provided the best fit to the data, with CFI and TLI values exceeding 0.95 and an RMSEA of less than 0.06.

**Table 3:** Comparison of fit indices among three proposed models

Fit indices	χ^2^	Df	*p* value	Chi/df	RMSEA	CFI	TLI	SRMR
Hypothesized-6 factor	521.019	155	0.000	3.361	0.085 (0.077–0.094)	0.903	0.881	1.254
Three-factor	248.516	133	0.000	1.869	0.052 (0.042–0.062)	0.969	0.956	0.044
One-factor	565.112	170	0.000	3.324	0.085 (0.077–0.092)	0.895	0.883	0.079

*df* degrees of freedom, *RMSEA* root-mean-square error of approximation, *CFI* comparative fit index, *TLI* Tucker-Lewis index, *SRMR* standardized root mean square residual

### Discussion

This study found that the Thai version of the IAT demonstrates similar satisfactory results to the previous study, particularly among the medical student sample. The corrected R squared of item 4 (‘forming new relationships with fellow on-line users’) presented the lowest value (0.179) of all items, which endorsed the findings of related studies in different cultures [12, 18, 22]. We hypothesized that this item may reflect the pathological behaviors associated with excessive Internet use less accurately than the remainder of the items.

In exploring factor structure, our results extracted three factors, whereas other researchers have proposed various factor solutions, ranging from 1 to 6. The first analysis of the IAT by two studies yielded a six factor-solution [28, 39]. Notably, both studies analyzed the test using a relatively low sample size (a sample of 86 in the former and 115 in the latter study). When repeated with a larger sample size, the number of factors was generally reduced to 2–3 [20, 22, 40]. This difference was likely due to the differences among the samples, which variously comprised college students and clinical samples, and whose cultural backgrounds differed.

Our findings differed from that of other research conducted among medical students. A Malay sample identified a five-factor model of the IAT [25], whereas a three-factor solution was found among Greek, Persian, and Pakistani samples [41–43]. Except for Malaysia, samples comprising medical students provided the same three-factor solution in various studies. Items within each factor differed due to the differences between the various samples’ characteristics, environments, and cultural and religious backgrounds.

Because no hypothesized model was proposed by Young in the original version of the IAT, attempts have been made to design more valid and reliable items in a newer version. In revising the IAT, many investigators have suggested removing the item(s) with low loading coefficients on designated factors or reducing problems with cross-loadings on other factors. However, these suggestions may be of limited relevance to the studied samples, and cannot necessarily be applied to other studies. One important property of the scale is its unidimensionality, which requires creating a cut-off score to define various levels of the problem. The unidimensionality of the model should be warranted to determine the legitimacy of summarizing the scale in a single score [44, 45]. Some investigators have suggested that, when the ratio of first-to-second eigenvalues is greater than four, the model can be considered unidimensional [46, 47].

To date, we have found only two studies identifying the IAT as a unidimensional model [13, 21]; one seems to show that it has sufficient unidimensionality [27]. The remainder of the existing studies found two or more factor solutions, including the present study. However, using the above criteria, the Thai version of the IAT showed sufficient unidimensionality. In addition, in the revising process, culturally biased items and differential item functioning due to sex should be identified to make the test more capable of being compared across cultures.

In conclusion, the Thai version of the IAT was shown to have good reliability and concurrent validity, as demonstrated by a significant correlation between the UCLA loneliness scale and the IAT. This relationship reflects some convergent validity because loneliness and Internet addiction were found to be correlated, especially among university or college students [31, 32]. A three-factor model fits the data well; in addition, it has sufficient unidimensionality to allow the total score to be used to screen for excessive Internet use.

### Limitations

This study has some limitations. First, even though the sample represents all levels of medical student, recruitment was not randomized. Second, test–retest reliability was not conducted to ensure temporal stability. Third, medical students may not be representative of the general population, so further examination of the validity and reliability of the test should be conducted in other populations. Further exploration in relation to shortening the scale to make it more unidimensional is encouraged.

## Additional file

**Additional file 1: Figure S1.** Eigenvalues among participants with Internet addiction. From the third component on, the line is almost flat, indicating each successive factor is accounting for smaller and smaller amounts of the total variance.

## Abbreviations

CFA: confirmatory factor analysis
CFI: Comparative Fit Index
DSM-5: diagnostic and statistical manual of mental disorders
EFA: exploratory factor analysis
IAT: internet addiction test
RMSEA: root-mean-square error of approximation
SRMR: standardized root mean square residual
TLI: Tucker–Lewis Index
UCLA: University of California Los Angeles
WLSMV: weighted least square means and variance adjusted
WRMR: weighted root-mean-square residual
