# Supplementary figures and images for: Psychometric properties of a Thai version internet addiction test
Source: BMC Res Notes. 2018 Jan 24;11:69. doi: 10.1186/s13104-018-3187-y (PMC5781275; doi:10.1186/s13104-018-3187-y)

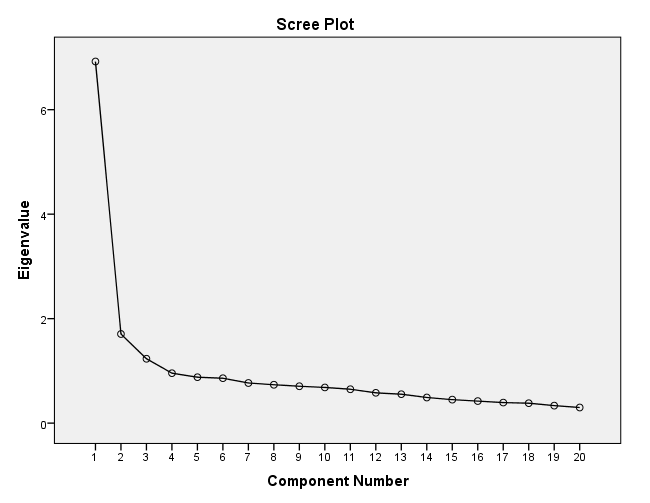

Supplement: Supplementary file 1 — Additional file 1: Figure S1. Eigenvalues among participants with Internet addiction. From the third component on, the line is almost flat, indicating each successive factor is accounting for smaller and smaller amounts of the total variance. [file 13104_2018_3187_MOESM1_ESM.tif]
